# Artistic craving in fronto-temporal dementia: escaping the tyranny of normality?

**DOI:** 10.1093/braincomms/fcag177

**Published:** 2026-06-22

**Authors:** Mira Didic, Francesca De Anna, Victor Altmayer, Lucas A Ronat, Olivier Felician

**Affiliations:** Assistance Publique–Hôpitaux de Marseille, Service de Neurologie et Neuropsychologie, Timone, 13005 Marseille, France; Aix-Marseille Univ, Institut des Neurosciences de Systèmes, Inserm U1106, 13005 Marseille, France; Assistance Publique–Hôpitaux de Marseille, Centre Constitutif Démences Rares et Précoces, 13005 Marseille, France; Assistance Publique–Hôpitaux de Marseille, Service de Neurologie et Neuropsychologie, Timone, 13005 Marseille, France; Assistance Publique–Hôpitaux de Marseille, Service de Neurologie et Neuropsychologie, Timone, 13005 Marseille, France; Aix-Marseille Univ, Institut des Neurosciences de Systèmes, Inserm U1106, 13005 Marseille, France; Sorbonne University, FrontLab at Paris Brain Institute (ICM), INSERM, CNRS, 75013 Paris, France; Centre de Recherche, Institut Universitaire de Gériatrie de Montréal, Montreal, Québec, Canada H3W 1W5; Department of Medicine, University of Montreal, Montreal, Quebec, Canada H3T 1J4; Department of Neurosciences, Faculty of Medicine, Université de Montréal, Montreal, Canada QC H3T 1J4; Department of Radiology, Radiation Oncology and Nuclear Medicine, Faculty of Medicine, Université de Montréal, Montreal, Canada QC H3T 1J4; Assistance Publique–Hôpitaux de Marseille, Service de Neurologie et Neuropsychologie, Timone, 13005 Marseille, France; Aix-Marseille Univ, Institut des Neurosciences de Systèmes, Inserm U1106, 13005 Marseille, France

## Abstract

Didic *et al.* describe the emergence of visual artistic abilities in frontotemporal dementia, moving beyond the traditional focus on dysfunction. Integrating the patient’s wife’s testimony with clinical, network-based and philosophical perspectives, they show how artistic production can become a mode of communication and challenge conventional boundaries between pathology and creativity.

***This essay explores the paradoxical emergence of visual artistic abilities in a patient with fronto-temporal dementia. Moving beyond the traditional focus on dysfunction, we highlight how behavioural changes can also unveil novel forms of self-expression, creativity, and meaning. To do so, the manuscript is partly presented through the perspective of the patient’s wife, whose testimony provides a deeply human account of how artistic activity became a mode of communication and connection. Drawing on her voice, complemented by clinical observations, network-based models, and philosophical perspectives on normality and pathology, we argue that artistic production in FTD challenges conventional boundaries between disease and creativity.***

Neurology has traditionally been a discipline of deficits. Patients with neurodegenerative diseases are usually looked upon from the angle of dysfunction. Assessments focus on loss of function as well as behavioural, cognitive and emotional disturbances: memory loss, language loss and loss of self. This approach leads to a negative impact on patients’ self-esteem, their caregivers and social integration. It also risks eclipsing something else: the possibility that within degeneration, paradoxical forms of expression may emerge. In fronto-temporal dementia (FTD), where inhibition and social convention collapse, a window sometimes opens onto unexpected creativity, defined as ‘the ability to produce a work that is both original (new, unexpected, unusual, novel) and valuable (useful, good, adaptive)’. Hence, although very rarely, heightened artistic pursuit can be observed in patients with FTD.^1^ However, both, the mechanisms that lead to these productions, and their artistic and creative versus pathological nature, remain a matter of debate. While many reports indicate that such patients provide a valuable opportunity to improve the understanding of artistic skill, its creative nature has been challenged with the proposal that some cognitive deficits may lead to ‘pseudo-creative’ behaviour.^2^ After observing a surge of visual artistic activity in a patient with FTD we propose a reconciling explanation. Also, observing the way in which his wife and family coped with his condition leads us to consider behavioural change in a broader perspective challenging the way in which we look upon patients with neurodegenerative disorders.

## A patient’s story—from Ana’s perspective

**‘**Patrick was 53 when I noticed the first changes. He was more restless, talking endlessly, often slipping into jokes with a sexual undertone. He devoured ice cream, drank more than before, and seemed to lose interest in personal care. The man I had known—attentive, gentle, professionally respected as a physiotherapist—was becoming someone unfamiliar. In the beginning, I thought that he was suffering from depression. I finally had to oblige him to see a neurologist.

**Figure 1:**
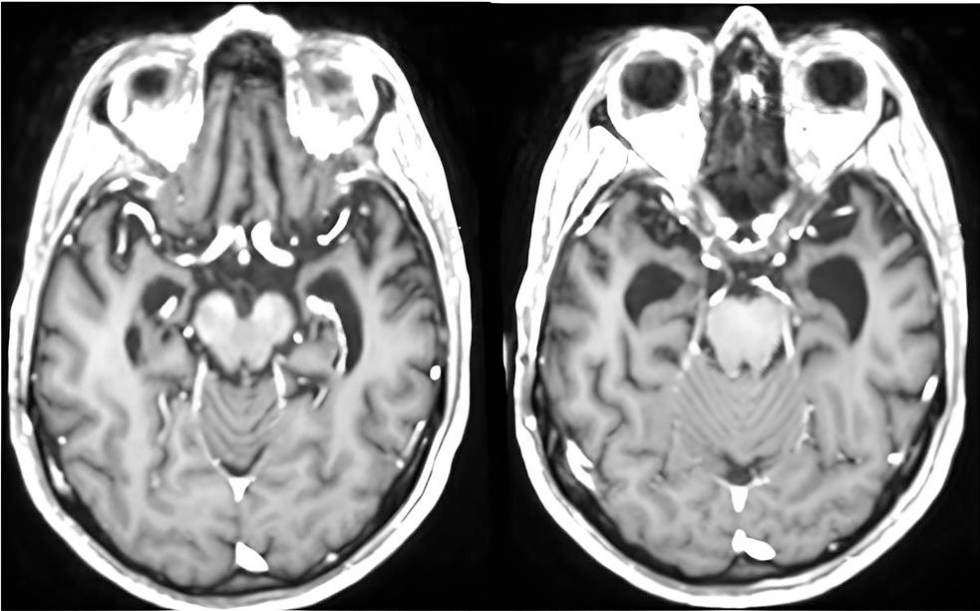
**Bilateral temporal atrophy on MRI.** Axial slice of T1-weighted MRI showing severe atrophy in the anterior temporal region bilaterally slightly predominating on the left (right side of the slide).

At the hospital, I watched the doctors, neuropsychologist and speech therapists examine him. His speech was fast, with innuendo and word play. They told me his knowledge of words was eroding, his executive functions faltering, yet his visual and spatial abilities were intact. Scans showed the atrophy in his anterior temporal lobes (Fig. 1), with hypometabolism on PET. The diagnosis came when he was 55: behavioural variant frontotemporal dementia. I knew, as a doctor myself, what this meant.

Just after the diagnosis, one of his patients accused him of inappropriate behaviour. The choc of legal procedures made us affront reality.

**Figure 2:**
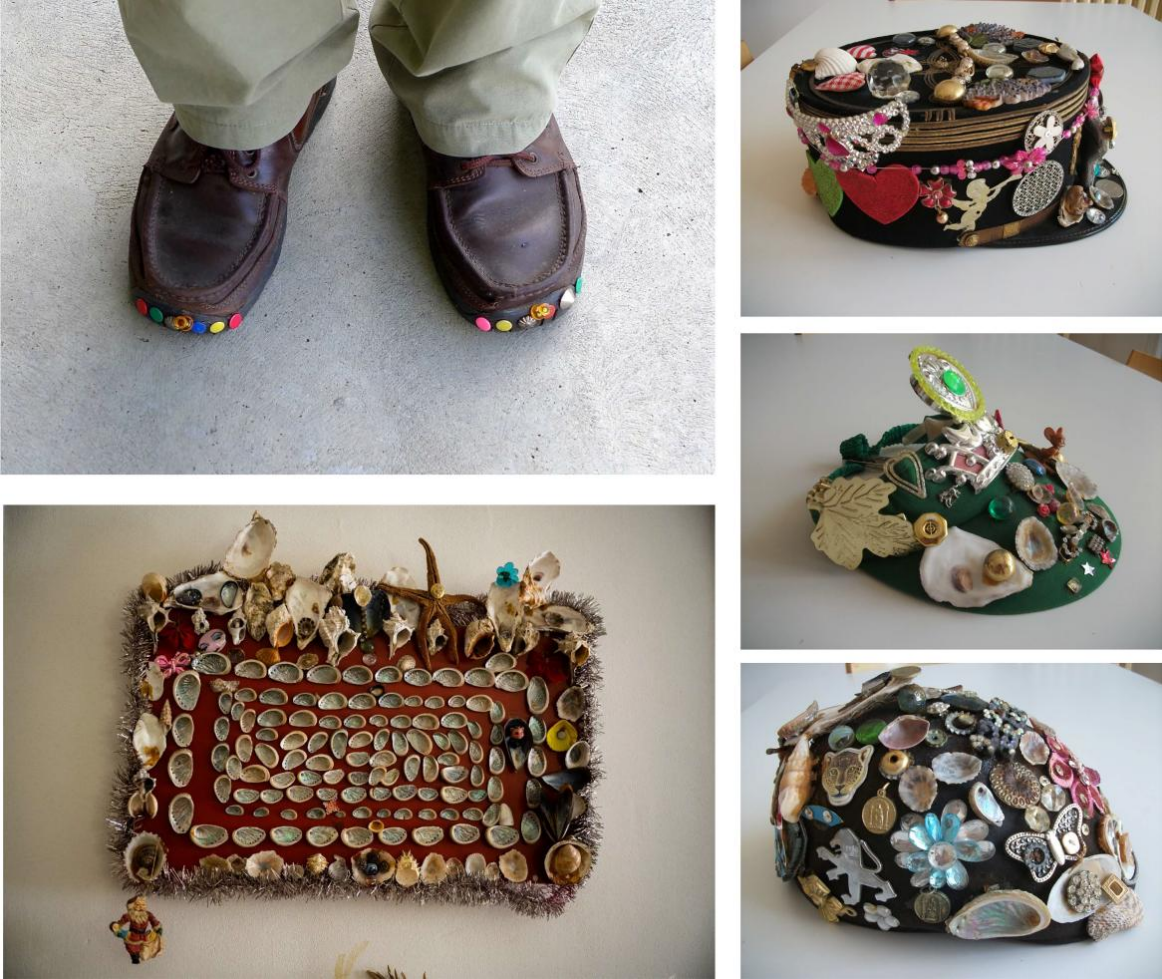
**Decorated hats and shoes.** Photograph of Patrick’s shoes (top left), of a collage on tissue (bottom left) and decorated hats (right).

But what unfolded in the years that followed was not only loss. He became flamboyant, eccentric, and sometimes egocentric. He began to dress in decorated hats and clothes that he transformed with his own hands (Fig. 2). Looking back, I realise this artistic turn had begun earlier, quietly. On long walks, he would collect shells, shiny fragments, objects abandoned on the street or the beach. At home, he stitched and glued them onto shoes, clothes, collages, even sewing a heart onto his favourite jacket. The objects were strangely beautiful. His creations filled our home. Neighbours saw him rummaging by bins, collecting pieces of metal, paper, shells. Some thought it shameful. I chose another path. ‘I do a lot of theatre,’ I told myself, ‘even if I never studied it. Because now I must play many roles: mother, daughter, wife, clown. I have to follow his current, not go against it—and learn how to guide it.’ It was in this spirit that his creativity became ours, a way to keep balance in a shifting life. I chose not to stop him but to accompany him. In them, I saw a part of him still striving to express himself.

Others were intrigued too. My cousin, a psychoanalyst, was so struck by Patrick’s creations that she made a documentary about him, together with a theatre director. The film—On va où, Ana?^3^—did not only show his decorated hats and collages; it also revealed how, through his illness, Patrick seemed to have escaped what we called the ‘tyranny of normality.’ It was as if the disease had dismantled conventions, opening space for another kind of truth. (Supplementary video 1 : teaser of ‘On va où, Ana’).

Nine years after the diagnosis, his body began to betray him too. Walking became difficult, his voice slurred, fasciculations marked his muscles. Tests confirmed motor neurone disease. Then came the cascade: more behavioural decline, mutism, paraparesis, a challenging Kluver–Bucy syndrome. In the end, he died of respiratory failure at 67. The autopsy confirmed what we had lived: widespread neuronal loss, the staining of TDP43 inclusions.

But what I hold on to are the creations: the hats, the shells, the collages. They remain as proof that even as the disease dismantled him, it also released something new’.

For us, the medical team, Patrick and Ana’s perspective was immensely instructive teaching us many lessons. It reminded us that medical examinations, neuropsychological assessments and scientific models explain only part of the story: much of what we understand about function and dysfunction in FTD comes from the lived wisdom of patients and their caregivers.

## Mechanisms of paradoxical creativity

The paradox of creativity in FTD has long intrigued neurologists. In terms of mechanisms, Miller *et al*.,^1^ while acknowledging the compulsive nature of artistic production in FTD, proposed that selective degeneration in anterior temporal and orbital cortex decreases inhibition on posterior areas like visual systems involved in perception, enhancing creative visual and artistic abilities. They also argued that loss of social skills and inhibition may have facilitated the art of their patients. Interestingly, they suggested that this new behaviour could be explained by ‘paradoxical facilitation’.^4^ Hence, while inhibitory and excitatory mechanisms are usually balanced or ‘in harmony’, the loss of inhibition by a lesion in one part of the brain could lead to unexpected, enhanced function of another part. More recently, a large case–control imaging study in patients with FTD identified 17 patients with visual creativity and suggested that activation of dorsal visual association areas induced by anterior atrophy could explain this behaviour.^5^ Interestingly, Ulugut *et al*.^6^ investigated changes in connectivity in the right temporal variant of FTD. This study found that reduced functional connectivity in the right ventral temporo-parietal network was linked to socioemotional-semantic deficits, while increased connectivity in the relatively spared right dorsal fronto-parietal network was associated with hyperfocus on fixed interests, or rigidity, enhanced artistic activity being the hyperfocus in some of the patients. Although this study did not specifically address artistic skill, it provides an experimental confirmation of Kapur’s ‘paradoxal facilitation’ hypothesis concerning the balance and interplay between damaged and spared networks in patients with hyperfocus on fixed interests.

## A complementary perspective within the framework of action control

Many authors have underlined the role of art in expressing the inner self, emotions, feelings and desire. We propose that this dimension can be usefully framed within the balance of ventral and dorsal systems of action control applied to several neurological and psychiatric diseases leading to behavioural dysfunction.^7^ Behaviours considered as ‘normal’ that guide our actions are based on a balance between internally guided cues (hedonic conditions as desire, including interoceptive inputs like autonomic processing) and externally guided cues (such as information coming from the environment like social rules and sensory experience). Two neural systems involved in action control have been identified: a medial system (in dorsal position), involved in self-initiated action and a lateral system (in ventral position) involved in actions initiated by others.^8^ Hence, in our patient, artistic activity may reflect a shift in network dynamics involved in action control: the ventral system, normally adapting behaviour to social cues, is impaired, while the dorsal system—more involved in self-initiated action—becomes disinhibited. The fact that the patient was overactive and displayed no signs of apathy is also in favour of this hypothesis. This imbalance could release internally generated impulses, allowing hedonic drives and interoceptive states to surface through creative production.

Such a mechanism echoes the idea of ‘paradoxical facilitation’^3^ and resonates with recent network models.^5^ Our proposal is also in line with findings that altered reward valuation leading to hedonic behavioural changes^9^ may contribute to emergent creativity, in addition to hyperactivation of dorsal visual association areas.^5^ Within a dynamic system, creativity in our patient, also referred to as the ‘hyperfocus on specific interests’ (or rigidity),^6^ could thus result from the change of balance, leading to a ‘release’ of the dorsal system involved in action control, resulting in an overexpression of internal hedonistic cues including interoceptive inputs expressing desire. Rather than contradictions, these perspectives suggest complementary pathways: degeneration may loosen external constraints while sparing or even amplifying internal drives that could be defined as ‘craving’, a strong uncontrollable desire, opening the way to unexpected forms of expression.

## Blurring normal and abnormal

Putting this into a broader perspective, we also question the limit between ‘pseudo-creative’ and creative behaviour, just as the limit between normal and pathological can be challenged. Within the framework introduced by Georges Canguilem in 1943 ‘*Could pathology be a change of the normal state?’* (‘*L’état pathologique n’est-il pas une modification de l’état normal?*’),^10^ we argue for a continuum rather than a limit between normal and abnormal. Within the theory of ‘*l’homme moyen*’ or the ‘*mean human being*’ of Adolphe Quetelet (1835),^11^ the norm is represented by the Gaussian curve, our reference in terms of performance, whether it concerns memory, reasoning, behaviour or social cognition. Within this continuum between normal and abnormal, many renowned artists symbolize this blur between normality and pathology. Hence, Vincent van Gogh’s productions, Ravel’s music and Dostojevsky’s writings may not have been conceivable without neural function beyond ‘the norm’. This conception reconciles the view expressed by Miller *et al*. (1995),^1^ that these patients express genuine artistic creativity with the claim that their productions results from ‘pseudo-creative’ repetitive and inappropriate behaviour.^2^ Hence, for us, there is no doubt that artistic activity in FTD can improve our understanding of what has been referred to as the creative process.

## Therapeutic and social dimensions

Beyond comprehension at the neural level, what is the deeper finality of this behavioural change? Yayoi Kusama, a renowned Japanese contemporary artist who suffers from mental problems stated ‘*I fight pain, anxiety, and fear every day, and the only method I have found that relieved my illness is to keep creating art, […] I followed the thread of art and somehow discovered a path that would allow me to live*’. This raises the question if some mechanisms of ‘self-healing’ could be involved not only in patients with psychiatric but also neurodegenerative disease. In this context, the social environment of our patient most certainly played an important role. The wife of the patient of the present report chose to accompany him in his creative drive leaving him considerable freedom of action. She tolerated socially inadequate behaviour like for example collecting objects in and around dustbins. They decided to accept the changes imposed by the disease together. This benevolence and encouragement of artistic production was fundamental as it promoted the patient’s well-being and became a mode of communication that enabled him to keep a link with his family and the outside world. Artistic activity in such patients appears to hold therapeutic value, fostering engagement in a pleasurable pursuit that offers scope for improvement. In this sense, we consider this activity as creative since the patient produced something both novel, useful and valuable, as it was therapeutic to him. Although sometimes on the edge of what we consider socially acceptable, or normal, artistic pursuit may enable some patients to escape the ‘*T*yr*anny of Normality*’.

## Supplementary Material

fcag177_Supplementary_Data

## Data Availability

The data underlying this article will be shared on reasonable request to the corresponding author.
