# Supplementary material for: Artistic craving in fronto-temporal dementia: escaping the tyranny of normality?
Source: Brain Commun. 2026 Jun 22;8(3):fcag177. doi: 10.1093/braincomms/fcag177 (PMC13305755; doi:10.1093/braincomms/fcag177)
Supplement: fcag177_Supplementary_Data [file fcag177_supplementary_data.zip › Supplementary video 1 caption.docx]

Supplementary Video 1. Teaser of the film “On va où, Ana”?
